# Telomere Length and Pediatric Obesity: A Review

**DOI:** 10.3390/genes12060946

**Published:** 2021-06-21

**Authors:** María Cristina Azcona-Sanjulian

**Affiliations:** 1 Paediatric Endocrinology Unit, Department of Paediatrics, Clínica Universidad de Navarra, Avenue Pío XII 36, 31008 Pamplona, Spain; cazcona@unav.es; 2 Institute of Research of Navarra, 31008 Pamplona, Spain

**Keywords:** obesity, overweight, telomere length, children, adolescents

## Abstract

Obesity is a chronic disease, which needs to be early detected early and treated in order prevent its complications. Changes in telomere length (TL) have been associated with obesity and its complications, such as diabetes mellitus and metabolic syndrome. Therefore, we conducted a systematic review to summarize results of studies that have measured TL in children and adolescents with obesity. Fourteen studies aiming to assess TL in pediatric patients with either obesity or who were overweight were included in this review. In conclusion, obesity and adiposity parameters are negatively associated with TL. Shorter telomeres are observed in children with obesity compared with their lean counterparts. Factors involved in obesity etiology, such as diet and physical activity, may contribute to maintenance of TL integrity. In the long term, TL change could be used as a biomarker to predict response to obesity treatment.

## 1. Introduction

Telomeres are sections of DNA located at the end of each of the chromosomes. They are nucleoprotein structures composed primarily of double strand repeats of TTAGGG followed by terminal 3′ G-rich single-stranded overhangs that protect the end of chromosomes, maintaining the stability of a genome [1]. They become shorter in each cell division and decline with age. Telomeres vary substantially among individuals from birth, and decrease with aging. The word telomere comes from the Greek, “telos” meaning end, and “meros” meaning part. The maintenance of telomere length is achieved by the enzyme telomerase, a reverse transcriptase, which preserves telomere length by adding telomeric repeats to the distal domains of re-synthesized linear chromosomes. Telomere length (TL) is heritable from the TL of the stem cells at birth, and is the result of a balance between TL shortening and TL maintenance that can be modified by several genetic, demographic and environmental factors [2].

TL in humans reflects variations in its initial length at birth as well as in the rate of telomere attrition throughout life, and it is an established biomarker of biological age. However, a high TL variation has been observed among individuals of the same or similar age in all age ranges evaluated [3]. In several twin studies, a high heritability of TL has been described [4]. Approximately 60% of interindividual TL variations and 30% of telomere age-dependent attritions are heritable [5]. Some studies have reported higher paternal heritability, however both paternal and maternal heritability have been demonstrated [6]. Moreover, longer TL is found in offspring conceived by older men [7]. Genetic variants also regulate TL and several genetic mutations (telomerase RNA and telomerase reverse transcriptase genes) involved in telomeres and telomerase are linked to telomere syndromes (dyskeratosis congenital and Werner syndrome) with premature aging disorders [8].

It is of utmost importance to identify factors that can modify positively TL dynamics and therefore promote health. In adults, short TL has been related to obesity [9], diabetes mellitus type 2 [10], hypertension [11], as well as to cigarette smoking, increased oxidative stress, younger paternal age, and to both psychological stress and affective disorders [12,13,14,15]. TL also depends on gender. Women have longer telomeres than men [16], however other studies do not find differences in TL with respect to sex [17]. Differences in TL have been found with respect to ethnicity, Afro-American adolescents having longer telomeres than Caucasian [18,19].

There are few studies, which analyse TL in pediatric populations with obesity, especially those assessing the effect of an intervention on TL. These studies are analysed further in discussion.

## 2. Materials and Methods

The systematic review was conducted following the preferred reporting items for systematic reviews and meta-analysis (PRISMA) statement [20].

### 2.1. Search Strategy and Eligibility Criteria

A literature search for all types of studies describing data on TL in children and adolescents who were either overweight or obese was conducted. The studies were considered eligible for their inclusion if they met the following criteria: (1) the participants were between 2 and 18 years, (2) the population of the study group were overweight/obese and associated metabolic diseases such as type 2 diabetes, insulin resistance, and cardiovascular risk factors. Studies that were not written in English or were grey literature without peer review processes, as well as reviews, editorials, opinions, letters and meeting abstracts were excluded.

### 2.2. Data Sources and Search Strategies

A systematic literature search in PubMed, selecting the original articles published from 2005 to January 2021, was performed. The key words used in the search strategy were related to the following topics: (1) subjects: children and adolescents, (2) telomere length (3) obesity and/or cardiometabolic outcome, (4) diabetes, insulin resistance.

The search strategy were as follows: (“children” OR “adolescent” OR “teenager” OR “boy” OR “girl”) AND (“obesity” OR “adiposity” OR “metabolic risk” OR “cardiometabolic risk” OR “type 2 diabetes” or “insulin resistance” OR “insulin sensitivity” OR “HOMA” (Homeostatic Model Assessment for Insulin Resistance)) AND (“telomeres” OR “telomere length” OR “TL change”).

The articles were imported to Mendeley data reference (version 1.19.8, Elsevier, USA). Duplicate fields were removed, firstly automatically by the software, and then by visual checking.

### 2.3. Study Selection Process

Six-hundred and five studies were identified through the database search. The titles and abstracts of these articles were examined to identify those that were likely to analyse TL in children and adolescents (≥12 years) with obesity, metabolic risk, cardiovascular disease, type 2 diabetes or insulin resistance.

**Figure 1:**
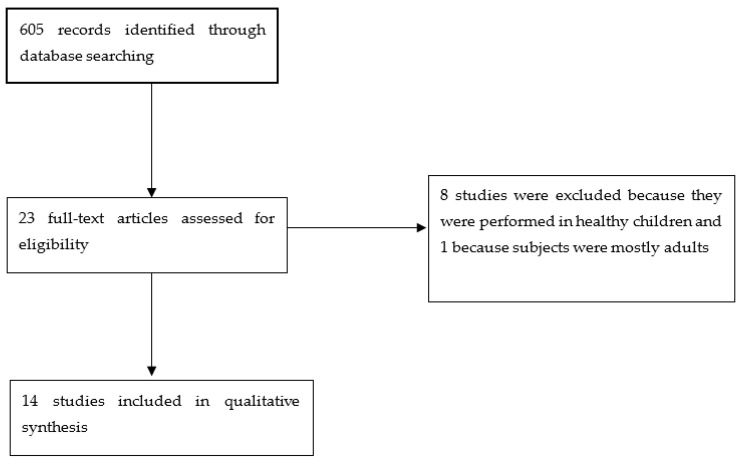
Flowchart of study selection.

Finally, 23 full text articles were assessed for eligibility; 8 were excluded because they were performed in healthy children and 1 was excluded because the subjects were mostly adults. Finally, fourteen studies were included (Figure 1). Those articles in which it was not possible to know their precise content by reading only the title and abstract were read in full, in order to decide the final inclusion or exclusion.

### 2.4. Data Collection Process and Data Items

The data from the included studies were extracted, and the data’s accuracy was checked. A specific database was created in Excel (Microsoft Corp, Redmond, WA, USA).

The following fields were collected from each included study: (1) study (author identification), (2) type of the study, (3) number of participants, (4) age of participants, (5) sex of participants, (6) sample and TL technique; and (7) main study conclusion.

### 2.5. Study Quality and Risk of Bias Assessment

Quality of the study was assessed using the systematical appraisal tool for cross-sectional studies, AXIS [21].

## 3. Results

### 3.1. Study Selection and Characteristics

Fourteen studies assessed the TL in children and adolescents who were either overweight or obese [22,23,24,25,26,27,28,29,30,31,32,33,34,35]. I was involved in 5 of the studies.

The characteristics of the study are described in Table 1. Most of the studies included adolescents (age ≥12years); there were only three studies which did not include them [22,29,31]. The distribution of boys and girls was similar in all the studies, at around 50%, and was not detailed in 3 of them [22,29,35]. In 3 studies, males were less than 50% (36% to 39%) [32,33,34].

The biological sample used was genomic DNA extracted from peripheral leucocytes in all of the studies except one, in which a saliva sample was used [34]. The method used to measure TL was a quantitative PCR in most of the studies; only one study that determined telomere restriction fragments (TRF) used another technique, described by Sotillo-Pinero et al. [36]. This technique requires a protein extraction step by CHAPS buffer (3-[(3-Cholamidopropyl)dimethylammonio]-1-propanesulfonate) followed by the telomeric repeat amplification protocol, which is explained elsewhere [37]. Finally, in 3 articles, a monochrome multiplex real time qPCR was used [32,33,34].

The participants were diagnosed with being overweight/obese according to their BMI status, and in 3 articles they were diagnosed with abdominal obesity using their waist circumference (WC) (percentile >90) [32,33,34]. Overweight was defined as when the BMI percentile was between 85 and 95, and obesity when the percentile was over 95.

The growth standards used to classify obesity were different in most of the studies. Five Spanish studies used the Spanish standard data [25,26,28,33,34]. Three studies, which were American, used the CDC growth charts [27,29,35]. One study performed in Arabic subjects applied the Cole et al. cut-off points [23]. One collaborative French and English study utilized the international reference for WHO [31]. A Greek study used the IOTF cut-off points [29]. One French study employed the French reference population [24]. An Italian study used the Italian cross-sectional growth charts [22].

Six of the studies were cross-sectional, two were case-controlled, and six were interventional studies.

### 3.2. Association between TL and Pediatric Obesity

Most of the studies found an association between shorter TL and either obesity or being overweight. This finding was described in very young children from 2 years of age [23]. There were only two studies, which did not find differences between obese and non-obese subjects. In one of these studies, they measured telomere restriction fragments, and this was the study with the smallest sample of subjects [22]. In the other study, the subjects were from different origins: European, American and African-American, and the samples to measure TL were taken from saliva [35].

In paediatric populations, there are few studies, which analyse the association between TL and obesity. Shorter telomeres were observed in children with obesity compared with their lean counterparts [23,30]. Lee et al. found a negative association between BMI and TL, which was stronger in younger subjects [38]. Clemente et al. observed an inverse association between TL and BMI-SDS, WC and skinfold thickness in children aged 6–11 years [31]. In contrast, no differences were found in Italian children either with or without obesity [22]. Moreover, no association between TL and adiposity parameters was observed in healthy adolescents aged 14–18 years [18].

### 3.3. Association between TL and Other Obesity Related Factors

In interventional studies, other interesting findings were described with respect to TL. García-Calzón et al. found that weight loss was accompanied by an increase in TL, and furthermore an initial longer TL could be a potential predictor for a better response to a multidisciplinary program (EVASYON: Development, implementation and evaluation of the efficacy of a therapeutic programme for adolescents with OW/OB: integral education on nutrition and physical activity (PA)) described previously [25,39]. The EVASYON is a multi-centre study conducted in 5 hospitals in 5 Spanish cities and two hundred and four OW/OB Spanish adolescents were recruited for a one year intervention. The adolescents were treated in groups of a maximum of 10 subjects; each group had 20 visits during the treatment period in two phases: intensive during the first 2 months (1^st^ to 9^th^ visits), and extensive during the last 11 months (10^th^ to 20^th^ visits). In order to assess the efficacy of the treatment, 8 dimensions were measured: diet; PA and fitness; eating behaviour; body composition; haematological profile; metabolic profile; minerals and vitamins; immuno-inflammatory markers.

García-Calzón et al., in an interventional study using a high antioxidant level diet, found that longer TL was associated with a higher dietary total antioxidant capacity as well as with a lower white bread consumption [26].

Zhu et al. observed in a large sample of subjects that a higher dietary sodium intake was associated with shorter TL in either overweight or obese 14–18 year-old adolescents [27].

Later on, García-Calzón et al. found in an interventional study (EVASYON study) that longer TL was associated with an improvement in fasting glucose levels and inflammatory markers after intervention [28]. In this line, Morell-Azanza et al. described in and intervention study that baseline TL could predict changes in fasting blood glucose levels after intervention [32].

Ojeda-Rodríguez et al., in an interventional study, found that favourable changes in diet quality indices could contribute to telomere integrity [33].

Furthermore, favourable changes in objectively measured PA were associated with longer TL in an intervention study in paediatric subjects with abdominal obesity [34].

## 4. Discussion

This systematic review aims to describe the main findings in paediatric obesity related to TL in either cross-sectional or interventional studies. There are not many studies regarding this topic, and especially very few interventional studies which assess the effect of a specific intervention on TL in paediatric patients with either obesity or who are overweight.

Most of the studies are cross-sectional, and the sample size is not large. The largest sample included 919 Greek children and adolescents aged nine to thirteen years. In particular, in this study, TL was diminished by 0.110 [30] with every increase in weight category (normal weight, overweight, obesity). In some studies, a difference in TL was found with respect to race, with African-Americans having longer TL than European-Americans [35]. The proportion of normal weight, overweight and obesity did not show significant differences with respect to race.

Some studies have evaluated the role of TL as a predictive biomarker for childhood obesity. In a cohort of Latino children, shorter TL in early childhood (four to five years) was a predictor for obesity at age nine [29]. On the other hand, longer TL predicted a higher decrease in body weight and BMI-SDS after a multidisciplinary program (EVASYON) in adolescents who were either overweight or obese [25]. On the contrary, a twelve-month randomized trial in adults, consisting of a dietary program and/or exercise training, found no significant changes in in TL, in postmenopausal women who were either overweight or obese [40].

Ojeda-Rodríguez et al. in an interventional study described that favourable changes in diet quality indices could contribute to telomere integrity [33]. Recently, a long-term randomized controlled intervention study analysed the influence of infancy-onset dietary intervention on TL; they found a small but statistically significant effect of the dietary and lifestyle intervention on the yearly attrition rate, but were unable to identify the specific intervention component underlying the observed effect [41].

In addition, favourable changes in objectively measured PA were associated with longer TL in an intervention study, in paediatric subjects with obesity [34]. In contrast, other previous studies reported a lack of association between PA and TL [42,43]. Regarding the intensity of PA, the findings are controversial. In the context of obesity, several exercise interventions assessed the possible role of PA on TL [40,44], but just one showed significant results [44]. Sjögren et al. found that a decrease in sitting time during weekdays was associated with longer TL in older adults who were overweight [43].

In some studies, a relationship has been described between TL and insulin resistance measured by a HOMA IR index, metabolic risk and inflammation markers. In an intervention study, a longer TL at baseline has been associated with a higher reduction in fasting glucose after two months of treatment [28]. Moreover, in a cross-sectional study, longer TL was associated with lower metabolic risk as well as with lower prevalence of high fasting glucose levels in children [45]. The metabolic risk in this study was assessed measuring some inflammatory markers such as C-reactive protein, fibrinogen, homocysteine and tissue plasminogen activator. Interestingly, in an intervention study, baseline TL could predict changes in fasting glucose levels [32]. The interventional study was a randomized control trial, in which the intervention group was treated with a hypocaloric Mediterranean diet and increase in PA, and the control group with the nutritional recommendations of the Nutrition Spanish society and increase in PA as well.

### 4.1. Strengths and Limitations

One of the main strengths of this review is that the studies are well designed and carried out. Moreover, all of the studies but one use the same sample to measure TL, and most of them utilize the same technique. However, the number of studies is small, as well as the sample size in most of them. Most of the studies apply different growth standards to diagnose being overweight/obese. Furthermore, few interventional studies have been performed.

### 4.2. Importance for Public Health

The measurement of TL could be used in the future as a potential biomarker of obesity, as well as an obesity treatment response marker. Nowadays, this use cannot be considered in a public health setting.

## 5. Conclusions

Obesity and adiposity parameters are associated with TL. Shorter telomeres are observed in children with obesity compared with their lean counterparts. Factors involved in obesity etiology such as diet and PA intervention in patients who are overweight or obese may contribute to maintaining TL integrity. Weight loss is accompanied by an increase in TL, and furthermore an initial longer TL could be a potential predictor for a better response to treatment. Longer TL is associated with an improvement in fasting glucose levels, and some inflammatory biomarkers after intervention. TL at the onset of an intervention could predict changes in weight reduction, improve fasting glucose levels, and lower inflammatory biomarkers after treatment.

## Figures and Tables

**Table 1:** Characteristics of the studies examining TL in obese children and adolescents.

Study	Settings	N	Age (Years) Mean ± SD or Range	Sex	TL Measurement and Sample	Conclusion
Zannolli 2008 [22]	Obese and normal children	53	8.2 (3.5)	Not detailed	TRF, DNA from PBMC	No difference in TL were found between obese and normal subjects
Al-Attas 2010 [23]	Cross-sectional study	148	5–12	46.6% male	PCR (IQcylinder) DNA from PBMC	Shorter TL in obese boys.
Buxton 2011 [24]	Case and control study	793	2–17	48% male	qPCR DNA from PBMC	Obese girls and boys have significantly shorter TL than their non-obese counterparts
García-Calzón 2014 [25]	Interventional study (EVASYON)	74	12–16	49% male	qPCR DNA from PBMC	Weight loss is accompanied by an increase in TL. Initial longer TL could be a potential predictor for a better response
García-Calzón 2015 [26]	Interventional study (GENOI)	287	6–18	55% male	qPCR DNA from PBMC	Longer TL were associated with higher dietary total antioxidant capacity and lower white bread consumption
Zhu 2015 [27]	Healthy obese and non-obese Afro-American adolescents	766	14–18	50% male	qPCR DNA from PBMC	Higher dietary sodium intake is associated with shorter TL in overweight and obese adolescents
García-Calzón 2017 [28]	Interventional study (EVASYON)	66	12–16	51% male	qPCR DNA from PBMC	Longer TL are associated with an improvement in glucose tolerance and inflammation after intervention
Kjaer 2018 [29]	Cohort Latino children	102	4–9	Not detailed	qPCR DNA from dried blood spots	Shorter TL in pre-schoolers associated with obesity at age 9.
Lamprokostopoulou 2019 [30]	Cross-sectional study Greek overweight/obese and normal children	919	9–13	50% male	qPCR DNA from PBMC	Shorter TL in overweight and obese subjects
Clemente 2019 [31]	European overweight/obese	1396	8 (1.5)	54% male	Modified qPCR DNA from PBMC DNA from PBMC	Shorter TL with higher adiposity indicators
Morell-Azanza 2020 [32]	Intervention study in abdominal obese children (IGENOI)	106	7–16	37% male	Monochrome multiplex real time qPCR DNA from PBMC	Inverse correlation between TL and obesity traits was observed in children with abdominal obesity. Baseline TL could predict changes in blood glucose levels
Ojeda-Rodríguez 2020 [33]	Intervention study in abdominal obese children (IGENOI)	87	7–16	39% male	Monochrome multiplex real time qPCR DNA from PBMC	Favourable changes in diet quality indices could contribute to telomere integrity
Ojeda-Rodríguez 2021 [34]	Intervention study in abdominal obese children (IGENOI)	121	7–16	36% male	Monochrome multiplex real time qPCR DNA from PBMC	Changes in physical activity had a direct effect on TL
Selvaraju 2021 [35]	Healthy obese and nonobese European American, African American children	127	6–10	Not detailed	qPCR DNA from saliva	No differences between obese and nonobese subjects

## Data Availability

Not applicable.
